# Intracranial Rescue Stenting in Pediatric Focal Cerebral Arteriopathy

**DOI:** 10.1007/s00062-025-01563-y

**Published:** 2025-09-09

**Authors:** Saujanya Rajbhandari, Philipe Breiding, Iciar Sanchez-Albisua, Daniel Brechbühl, Sandrine Cornaz Buros, Gabriela Oesch Nemeth, Johannes Kaesmacher, Lorenz Grunder, Petra Cimflova, Eike Piechowiak, David Seiffge, Maja Steinlin, Jan Gralla, Andrea Klein, Tomas Dobrocky

**Affiliations:** 1 Department of Diagnostic and Interventional Neuroradiology, Inselspital, Bern University Hospital, University of Bern, Bern, Switzerland; 2 Division of Neuropaediatrics, Development and Rehabilitation, Department of Paediatrics, Inselspital, Bern University Hospital, University of Bern, Bern, Switzerland; 3 Department of Neurology, Inselspital, Bern University Hospital, University of Bern, Switzerland Bern,

**Keywords:** Pediatric, Stroke, Infarction, Arteriopathy, Stent

## Abstract

**Background:**

Pediatric acute ischemic stroke is a rare yet severe condition with multifactorial etiology, often associated with vasculopathies. Endovascular intervention in children with focal cerebral arteriopathy is seldom reported.

**Purpose:**

Our aim was to report feasibility of intracranial rescue stenting for the management of pediatric focal cerebral arteriopathy with flow-limiting stenosis.

**Methods:**

We report a toddler with acute ischemic stroke due to flow-limiting focal cerebral arteriopathy of the left middle cerebral artery, treated with intracranial stenting. A comprehensive literature search was conducted in PubMed, Web of Science, Embase, and Scopus to identify case reports and series involving pediatric patients with acute ischemic stroke treated with intracranial stenting. Six cases met inclusion criteria. Extracted data included demographics (age, sex), clinical presentation, time of onset, medical history, occlusion location and etiology, stent type, pre- and post-stent NIHSS scores, antiplatelet therapy, and clinical outcomes at follow-up.

**Results:**

A total of six pediatric acute ischemic stroke cases with intracranial stent deployment in the acute stage were analyzed. The supraclinoid internal carotid artery was the most common site of stent deployment (4/6), while intracranial dissection was the most frequent cause of vessel occlusion (3/6). All included patients achieved resolution of the initial neurological deficit on follow-up (range: 6 weeks to 6 months). Variation in the use of intraoperative and postoperative antiplatelet regimens was observed.

**Conclusions:**

This case demonstrates off-label rescue stenting in pediatric acute ischemic stroke due to focal cerebral arteriopathy, emphasizing the importance of individualized multidisciplinary management in this rare setting.

## Introduction

Acute ischemic stroke (AIS) affects approximately 1 · 3–1 · 6 per 100,000 children annually in high-income countries. Around 70% of affected children experience long-term neurological deficits, significantly impacting their quality of life. Unlike adult stroke, the etiology of childhood stroke is distinct and frequently multifactorial, with cardioembolic events and vasculopathies, such as focal cerebral arteriopathy (FCA) being the most common causes [1].

According to the VIPS study (Vascular Effects of Infection in Pediatric Stroke), FCA is defined as a unifocal and unilateral stenosis/irregularity of the large intracranial arteries predominantly affecting the distal internal carotid artery (ICA) and its proximal branches [2]. FCA is classified into three subtypes: FCA-inflammation type (FCA-i), associated with inflammatory or post-infectious origins; FCA-dissection type (FCA-d), often linked to trauma; and undetermined FCA [3]. Children with arteriopathy have a high risk (> 50%) of recurrent stroke [1]. Although conservative management with antiplatelet agents or steroids has been described as an effective treatment for AIS secondary to FCA [4], endovascular therapy can be considered for selected patients with severe flow impairment. We present a case of a 2-year-old child diagnosed with severe flow-limiting left middle cerebral artery (MCA) stenosis secondary to FCA, successfully treated with acute intracranial stenting. We also present a systematic review of the literature, synthesizing data from similar pediatric cases to contextualize this therapeutic approach.

## Material and Methods

### Case Presentation

A 2-year-old child presented with a stumbling gait and repeated falls, which became apparent at 3 pm at a daycare center. The child had a history of normal development with no prior trauma. Apart from two days of fever, cough, and rhinorrhea preceding the presentation, there was no significant medical history. Upon clinical examination at an external hospital, the child exhibited right-sided hemiparesis and facial nerve palsy, with a Pediatric National Institute of Health Stroke Scale (PedNIHSS) score of 20.

**Fig. 1:**
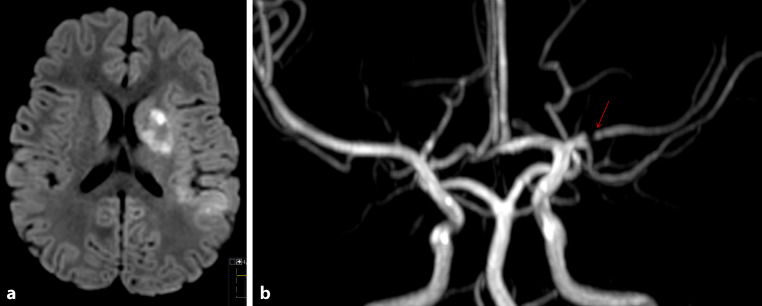
Axial diffusion-weighted image (**a**) demonstrates an infarct in the left basal ganglia. Time-of-flight angiography shows severe stenosis of the left proximal M1 segment (**b**, arrow)

The patient was immediately transferred to a comprehensive stroke center. Brain magnetic resonance imaging (MRI) demonstrated a left MCA territory stroke with diffusion-restriction in the basal ganglia and a small cortical infarct in the precentral area with corresponding subtle hyperintensity on fluid-attenuated inversion recovery (FLAIR) imaging (Fig. 1a).

**Fig. 2:**
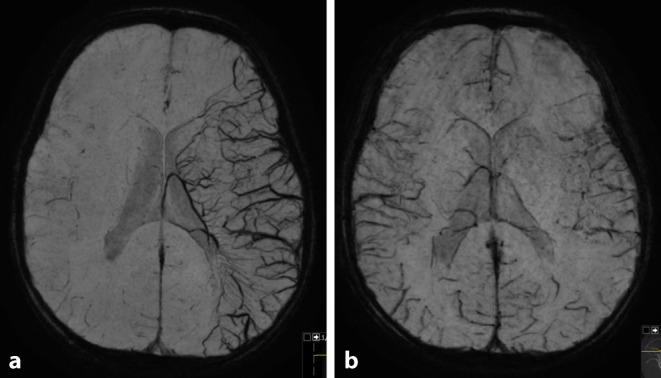
Baseline susceptibility-weighted image (SWI) shows prominent veins in the left middle cerebral artery territory and paucity of veins in all other vascular territories (most likely due to mechanical ventilation with O2) (**a**). Normalization of veins post-operatively with symmetrical vascular pattern (patient was slightly sedated but not intubated for the exam) (**b**)

**Fig. 3:**
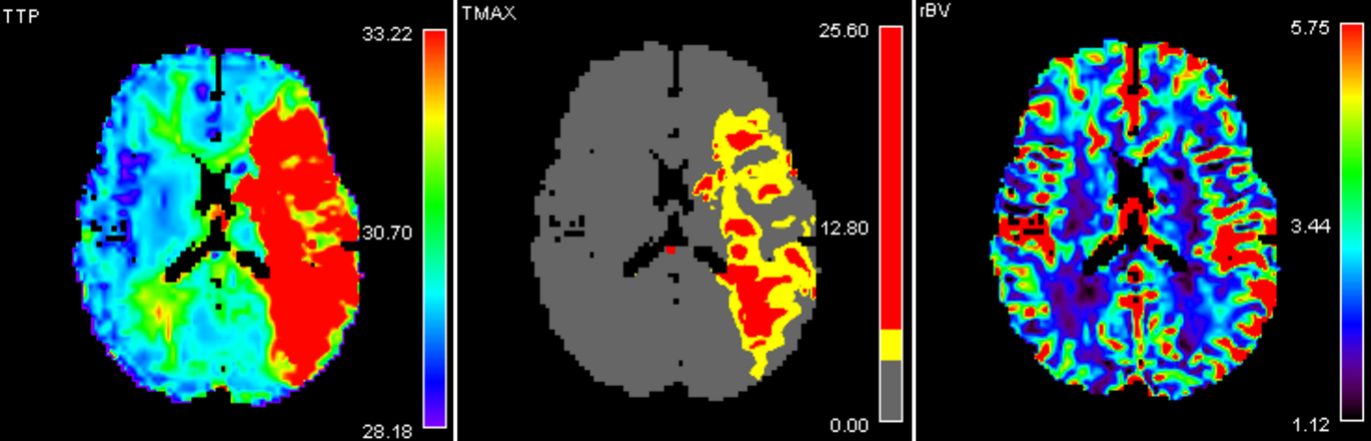
MRI Time-to-peak (TTP, right) and Tmax (middle) perfusion maps reveals a severe delay in the entire left middle cerebral artery territory with preserved cerebral blood volume (CBV, left)

On arterial Time-of-flight (aTOF) magnetic resonance angiography (MRA), severe unifocal stenosis in the left proximal M1-MCA segment, just distal to the origin of the anterior temporal branch, was apparent (Fig. 1b). Focal Cerebral Arteriopathy Severity Score (FCASS) was rated as 3 (stenosis > 50%) [2]. Susceptibility-weighted image (SWI) demonstrated striking prominence of intracranial veins in the entire left MCA territory (Fig. 2a, b). Perfusion-weighted imaging (PWI) demonstrated a corresponding hypoperfusion within the entire left MCA territory (Fig. 3). Vessel wall imaging (VWI) did not demonstrate any wall enhancement.

Intravenous thrombolysis with alteplase was initiated (0.9 mg/kg). Given the severe stenosis of the left M1 segment and a large diffusion-perfusion mismatch, after counseling the parents the decision to proceed with endovascular treatment was made by the multidisciplinary team. Symptom onset to groin puncture time was 6 h.

**Fig. 4:**
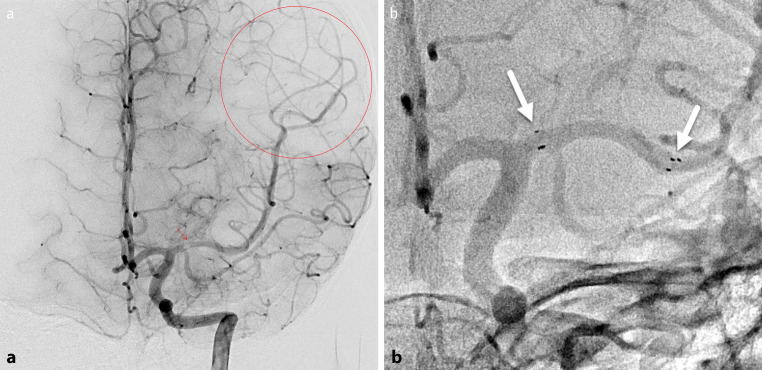
Initial left internal carotid artery (ICA) digital subtraction angiography (DSA) demonstrates severe focal stenosis of the middle cerebral artery M1 segment (**a**, arrow). Note the delay in the capillary phase of the left middle cerebral artery territory (circle) compared to the surrounding parenchyma supplied by the unaffected anterior temporal branch and the anterior cerebral artery (**a**). Post-stenting DSA run demonstrates no residual stenosis (**b**, arrow)

The procedure was performed under general anesthesia. Under ultrasound guidance a 4-Fr sheath was placed in the right common femoral artery and cerebral diagnostic angiography was performed using a 4 Fr catheter (Vertebral; Cordis, Florida, USA). Left ICA digital subtraction angiography (DSA) showed a circumscribed filling defect in the proximal M1 segment and confirmed a flow-limiting stenosis with venous delay (> 2 sec) in the downstream MCA territory (Fig. 4a). A microcatheter (Sl-10; Stryker Neurovascular, Fremont, California, USA) was navigated distal to the lesion, and one thrombectomy maneuver with a stent-retriever (Catch-mini, 3 × 15 mm; Balt, France) was performed. During the withdrawal of the stent retriever, manual aspiration was applied via the 4 Fr guide catheter in the ICA, using a 50 cc VacLok® syringe. No thrombus was present in the stent struts nor the aspiration syringe. The control run demonstrated a linear appearing filling defect in the proximal M1 segment suggestive of an intimal flap with a persistent delay in the MCA territory. Given that FCA was considered the most likely underlying etiology, percutaneous transluminal angioplasty (PTA) was withheld to minimize the risk of vessel rupture.

Following intravenous administration of a weight-adapted bolus of aspirin (3 mg/kg—total: 50 mg), a self-expanding, low-profile stent with anti-thrombogenic Hydrophilic Polymer Coating (pEGASUS 3.5 × 15 mm; Phenox GmbH, Bochum, Germany) was deployed across the lesion in the M1 segment, with the proximal end landing just distal to the carotid‑T. After stent deployment, there was immediate flow improvement and no residual stenosis. The final DSA run 20 min after stent deployment demonstrated no thrombus formation and regular antegrade filling of the MCA territory (Fig. 4b). After catheter- and access sheath removal, the femoral puncture site was manually compressed to achieve hemostasis. The patient was extubated in the angiography suite and transferred to the pediatric intensive care unit for post-interventional surveillance. The child was transitioned to oral aspirin (3 mg/kg) daily the next morning. A follow-up MRI on day one, demonstrated no new DWI lesions, complete resolution of prominent veins in SWI (Fig. 2b), and no hypoperfusion in the MCA-territory. There was no hemorrhagic transformation in the previously delineated ischemic areas (Fig. 2b).

A nasopharyngeal swab tested positive for parainfluenza and rhinovirus. The post-interventional course was uneventful, with the PedNIHSS score improving from 20 to 0 by discharge on day 10. At the 6‑week follow-up, no new neurological deficits were observed. Follow-up MRI revealed subtle gliotic changes of the affected areas in left MCA territory, with no evidence of new infarcts or new intracranial stenosis.

### Literature Review

The institutional review board has waived the need for an ethical approval since the current study involved the analysis and synthesis of previously published data. We adhered to the Preferred Reporting Items for Systematic Reviews and Meta-Analyses (PRISMA) 2020 statement for conducting this systematic review study [5]. The literature search, duplication removal, full-text article screening, and data extraction were all done using the Autolit platform (Nested Knowledge, St. Paul, Minnesota, USA). To identify all relevant manuscripts four major databases including PubMed, Web of Science, Embase, and Scopus were searched. The search strategy involved a combination of keywords and subject headings related to (“pediatric” OR “child”) AND (“acute ischemic stroke” OR “cerebral infarction”) AND (“thrombectomy” OR “stent”).

References from previous studies were also reviewed. We included all case reports and case series involving pediatric patients with acute ischemic stroke who underwent intracranial stenting. We collected demographic (age, sex), clinical data (symptom, time of onset, medical history, location of occlusion, cause of occlusion), type of intracranial stent, pre-stent and post-stent NIHSS, clinical outcome (follow-up) and antiplatelet regimen.

## Results

Literature review identified six pediatric AIS cases with intracranial stent deployment in the acute stage (Table 1; [6–9]). The most frequent site of stent deployment was in the supraclinoid ICA (4/6) and the most frequent cause of vessel occlusion was intracranial dissection (3/6). All included patients achieved resolution of the initial neurological deficit on follow-up (range: 6 weeks to 6 months). Variation in the use of intraoperative and postoperative antiplatelet regimens was observed. The antiplatelet agents administered included aspirin, clopidogrel, and tirofiban.

**Table 1:** Summary of reports involving pediatric patients with acute ischemic stroke who underwent intracranial stenting.

Author and year	Age	Sex	Symptom	Time of onset	Medical history	Location of stenosis	Cause of occlusion	Intracranial stent	Pre-stent NIHSS	Post-stent NIHSS	Clinical outcome (Follow-up)	Antiplatelet regimen
Present study	2 years	M	Right hemiparesis and right facial palsy	8 h	Parainfluenza and rhinovirus infection	Left M1 segment	Focal cerebral arteriopathy	3.5 × 15 mm pEGASUS (Phenox GmbH, Bochum, Germany)	20	0	At 6 weeks follow-up: No new neurological deficit	Intraoperatively: Intravenous aspirin (3 mg/kg—total: 50 mg); Next day: Transitioned to oral aspirin (3 mg/kg) daily
Rezayev et al., 2024 [6]	10 years	F	Right hemiparesis and aphasia	8 h	Trauma	Left supraclinoid ICA	Dissection	3 × 24 mm Neuroform Atlas Stent (Stryker Neurovascular, Fremont, California, USA)	20	NA	At 5 months follow-up: complete resolution of dysarthria and extremity weakness	Intraoperatively: 75 mg of clopidogrel and 325 mg of aspirin;
											At 1‑year post-stent placement, patient had no motor deficits and mild cognitive issues	After 24 h to 6 months: 37.5 mg of clopidogrel and 81 mg of aspirin daily;
												After 6 months: 81 mg of aspirin daily indefinitely
Jay et al., 2021 [7]	Adolescent	NA	Right hemiparesis and aphasia	Deteriorated on hospital day 2	Heterozygous mutation in FKBP14 gene	Left supraclinoid ICA	NA	4 × 20 mm Neuroform EZ stent (Stryker Neurovascular, Fremont, California, USA)	14	1	At 6 months follow-up: full-strength recovery and some subtle speech hesitancy (mRS: 0)	Intraoperatively: Tirofiban (25 mcg/kg); Next day: transitioned from tirofiban (0.15 mcg/kg/min over 18 h) to aspirin 325 mg daily and clopidogrel (300 mg load then 75 mg orally daily)
Jay et al., 2021 [7]	Adolescent	NA	Right hemiparesis and aphasia	Deteriorated on hospital day 6	Attention-deficit disorder	Left supraclinoid ICA	Dissection	4.5 × 30 mm Neuroform EZ stent (Stryker Neurovascular, Fremont, California, USA)	11	2	At 6 months follow-up: Neurologically intact (mRS:0)	Intraoperatively: Tirofiban (25 mcg/kg); Next day: transitioned from tirofiban (0.15 mcg/kg/min over 18 h) to aspirin 325 mg daily and clopidogrel (300 mg load then 75 mg orally daily)
Yu et al., 2012 [8]	4 years	F	Left hemiparesis and left facial palsy	1.5 h	Complex heart disease	Right distal ICA	NA	4.5 × 28 mm Enterprise (Cordis Neurovascular, Inc., Miami, FL, USA)	10	NA	At 6 months follow-up: No new neurological sequele	Aspirin and warfarin
Binning et al., 2010 [9]	14 years	M	Left hemiparesis and left facial palsy	NA	Trauma	Right supraclinoid ICA and MCA	Dissection	4.5 × 28 mm Enterprise (Cordis Neurovascular, Inc., Miami, FL, USA)	NA	NA	NA	Intraoperatively: loading dose of aspirin (650 mg) and clopidogrel (600 mg); After 24 h to 6 months: 325 mg of aspirin and 75 mg of clopidogrel; After 6 months: Aspirin monotherapy

*ICA* internal carotid artery, *MCA* middle cerebral artery, *NA* not available

## Discussion

To our knowledge, this is the first reported case of pediatric AIS due to FCA‑i treated with intracranial rescue stenting in the acute stage, achieving immediate flow restoration and complete clinical improvement.

FCA accounts for 20–26% of pediatric strokes in Europe and 2–13% in North America. It is often monophasic, showing rapid initial progression over days to weeks, stabilizing at a non-progressive plateau within six months, with some cases resolving completely [10]. Conservative management including steroids might be a viable option in FCA‑i to prevent progressive arterial stenosis and occlusion. However, in cases of severe flow-limiting stenosis, the benefits of such treatment may be insufficient [4]. This underscores the importance of identifying patients who are likely to benefit from endovascular intervention.

Multiple randomized controlled trials have provided the highest level of evidence and established mechanical thrombectomy (MT) as a mainstay of AIS treatment in adults, however, the evidence in the pediatric population remains scarce. The Save ChildS Study, a retrospective, multicenter registry, included 73 children with AIS undergoing MT between 2000 and 2018. The authors reported excellent rates of favorable clinical outcome (median mRS score at discharge 1.0 [IQR, 0.2–2.0]) and similar reperfusion rates compared to adults (complete reperfusion ≥ mTICI 2b in 62 of 71 patients [87%]) [11]. However, in the KidClot study, arterial reocclusion or stenosis ≥ 50% was more frequent in patients with FCA (5 of 7 [71.4%]) vs cardioembolic (CE) and dissection-related stroke (3 of 13 [23.1%]) on 24 h follow-up imaging after MT [12]. A pooled analysis of the Save ChildS and KidClot cohorts, included 60 children undergoing MT, 14 with FCA, and 46 with CE origin. FCA was associated with significantly lower rates of excellent revascularization (21% vs 65%, *p* < 0.001). [11–13] The recently published Save ChildS Pro Study, included 208 children (117 undergoing MT, and 91 receiving best medical treatment) between 2020 and August 2023 [14]. The authors reported no difference in median mRS at 90 d for patients with suspected FCA treated with MT (*n* = 18) and best medical treatment (*n* = 33) (2 [IQR 1–3] vs 2 [1–4]; *p* = 0 ∙ 074). However, there was an improvement in secondary outcomes in the MT group with improved PedNIHSS at discharge, 90-day Pediatric Stroke Outcome Measure, safety outcomes like intracranial hemorrhage, and 90-day mortality. The results support the benefit of MT in severely affected patients with suspected FCA; whereas patients presenting with milder stroke due to FCA might not benefit in the same way [14].

The pathophysiological differences of pediatric stroke, especially between FCA and CE, are a likely explanation for differences in reperfusion rates and merit closer consideration. In general, all currently used mechanical thrombectomy techniques (aspiration, stent-retriever, combined approach) are likely to be effective in the presence of a clot irrespective of the patient’s age. However, given the strikingly low rates of complete reperfusion in FCA and the high rates of reocclusion, rendering the endovascular procedure futile in a considerable number of children, choosing the appropriate endovascular tool and considering rescue technique seems crucial. Despite the widespread notion of increased vessel wall fragility, no cases of subarachnoid hemorrhage in the setting of FCA‑i have been reported in the literature.

In our patient, there was no history of trauma that would suggest an isolated traumatic MCA dissection. Due to the recent history of viral infection, FCA‑i was considered the most likely cause. FCA‑i is thought to be due to an inflammatory process of the affected vessel wall segment, leading to luminal narrowing or thrombus formation on inflamed and damaged endothelium. The initial DSA showed a focal filling defect resembling a clot, prompting a thrombectomy attempt. However, this maneuver did not achieve successful recanalization. We concluded that further thrombectomy attempts were unlikely to recanalize the vessel and might increase the risk of complications. Due to a persistent severe flow-limitation and a large downstream territory at risk, we opted for rescue stenting.

There is a paucity of data on intracranial rescue stenting in acute ischemic stroke in the pediatric population. Rezayev et al. reported a likely post-traumatic, flow-limiting supraclinoid ICA dissection in a 10-year-old girl, which was managed with stenting (Table 1; [6]) The patient was maintained on daily aspirin (81 mg) and Plavix (37.5 mg) for 6 months and has achieved a very good clinical recovery [6]. Jay et al. reported two adolescent patients with supraclinoid ICA dissections treated with acute stenting (Table 1; [7]) Both patients were transitioned to daily aspirin (325 mg) and Plavix (75 mg) and made a recovery to a modified Rankin Scale (mRS) of 0. [7] In 2012 Yu et al. reported a 4-year-old girl with a complex congenital heart disease, presenting an intracranial ICA occlusion, who underwent rescue stenting after thrombolysis and balloon-angioplasty failed (Table 1; [8]).

Generally, deployment of an intracranial stent in the acute phase requires effective antithrombotic treatment and may increase the risk of intracranial hemorrhage. In the reported case, due to established infarct core on baseline imaging, we aimed to avoid dual antiplatelet therapy to minimize the risk of hemorrhagic transformation. The stent deployed in our patient was polyethylene glycol based Advanced Surface Using Synthesis (pEGASUS, Phenox GmbH, Bochum, Germany) which features a covalently bound hydrophilic polymer coating on the stent struts that reduces thrombogenicity. The stent has been approved for the treatment of aneurysms and intracranial stenosis since 2021. Pielenz et al. reported the feasibility of pEGASUS-HPC stent system in 41 adult patients (with a mean age of 71 years) with intracranial stenosis in the setting of acute ischemic stroke (as the rescue treatment due to adherent thrombus, atherosclerotic disease, or dissection) as well as in elective cases. The authors reported a mean stenosis improvement of 53.0 ± 18.0%. [15] However, data from this adult cohort cannot be directly translated to pediatric patients.

Regarding the antiplatelet regimen, we administered an intravenous weight-adapted bolus of aspirin (50 mg) before stenting. Following the procedure, no thrombus formation was observed on the repeated DSA (20 min after stent deployment), so no additional antiplatelet medication was administered, and we transitioned to oral aspirin (3 mg/kg) the next day. Variations in the antithrombotic treatment exist in reviewed pediatric AIS cases after stenting (with no surface modification of the used stents), with most protocols recommending dual antiplatelet therapy for the first 24 h. Many centers subsequently transitioned to single antiplatelet therapy with aspirin after six months. Commonly used antiplatelet agents were aspirin, clopidogrel and tirofiban.

Rescue stenting may serve as a therapeutic option for selected FCA cases with flow-limiting stenosis, particularly when symptomatic exacerbation is a concern. The study has several limitations, including the small sample size and the heterogeneity of the reported cases, which makes direct comparisons difficult. A potential limitation in paediatric stenting is uncertainty about long-term outcomes, particularly in relation to vessel growth. We expected minimal further diameter increase that would impact stent function, as craniocervical vessels in children aged 1–2 years reach nearly 80% of their adult diameter [16]. The study underscores the need for further research into tailored endovascular strategies for pediatric stroke and may inform future clinical guidelines by expanding the range of treatment options for this vulnerable population.

## Conclusion

This case highlights the multidisciplinary approach in the management of pediatric FCA with flow-limiting stenosis, achieving complete recanalization through intracranial rescue stenting. Although rescue stenting in paediatric AIS is off-label, it should be considered for selected cases with flow-limiting stenosis (e.g., due to FCA) who are at risk of clinical deterioration and recurrent ischemic events.
